# Introduction to the Special Issue “Skeletal Muscle Atrophy: Mechanisms at a Cellular Level”

**DOI:** 10.3390/cells12030502

**Published:** 2023-02-03

**Authors:** Emanuela Zuccaro, Caterina Marchioretti, Marco Pirazzini, Maria Pennuto

**Affiliations:** 1 Department of Biomedical Sciences (DBS), University of Padova, 35128 Padova, Italy; 2 Veneto Institute of Molecular Medicine (VIMM), 35128 Padova, Italy; 3 Padova Neuroscience Centre (PNC), 35128 Padova, Italy; 4 Cir-Myo, Centro Interdipartimentale di Ricerca di Miologia, University of Padova, 35131 Padova, Italy

**Keywords:** muscle atrophy, muscle proteostasis and disuse, atrogenes, sarcopenia, neuromuscular disorder, myopathies, muscle degeneration, neuromuscular paralysis, cancer cachexia

## Abstract

Skeletal muscle is the most abundant tissue in the body and requires high levels of energy to function properly. Skeletal muscle allows voluntary movement and body posture, which require different types of fiber, innervation, energy, and metabolism. Here, we summarize the contribution received at the time of publication of this Introductory Issue for the Special Issue dedicated to “*Skeletal Muscle Atrophy: Mechanisms at a Cellular Level*”. The Special Issue is divided into three sections. The first is dedicated to skeletal muscle pathophysiology, the second to disease mechanisms, and the third to therapeutic development.

## 1. Introduction

In humans, skeletal muscle is the most abundant tissue, comprising approximately 40% of total body weight and 50–75% of all body proteins. Muscles are under the control of the somatic nervous system to carry out any voluntary movements, from the most basic ones, such as breathing, to the finest and most complex ones, such as flying for a bird or painting for a human. For this reason, skeletal muscle is one of the most dynamic and plastic tissues in the human body, which continuously adapts to internal and external stimuli. Muscle mass is among the most relevant factors of this adaptation and, not by chance, often becomes a parameter to assess, even visually, the overall health or disease status of individuals. It depends on a complex balance between protein synthesis and degradation. These processes are regulated by different factors, such as diet, hormone levels, physical activity, and injuries or disease conditions. The principal function of skeletal muscle is to convert electrochemical energy into mechanical work to generate force and power for body movement. Skeletal muscles allow for a wide variety of voluntary movements, starting from continuous low-intensity activity (posture maintenance) to repeated submaximal (walking) and strong maximal contractions (jumping) [1]. Several conditions, from physiological aging to pathological conditions such as neuromuscular disorders, neurodegenerative diseases, and cancer, can lead to loss of muscle mass and force production, resulting in muscle atrophy. Tremendous efforts led to a better understanding of the mechanisms underlying muscle atrophy, but much remains to be clarified at the molecular and cellular levels. A wide range of cellular (e.g., myofibers and satellite cells) and subcellular (e.g., neuromuscular junctions) compartments, organelles (e.g., mitochondria and sarcoplasmic reticulum), degradation pathways (e.g., ubiquitin-proteasome system and autophagy), molecular signaling networks (e.g., AKT, mTOR, etc.), and genes (e.g., atrogenes) have been identified, which serve as critical players in the regulation of muscle mass and atrophy and become critical factors in the plasticity and vulnerability of muscle tissue under physiological and pathological conditions [2]. In this Special Issue of Cells entitled “*Skeletal Muscle Atrophy: Mechanisms at a Cellular Level*”, we present a collection of reviews and research articles focused on the cellular and molecular mechanisms responsible for muscle atrophy, which can ultimately lead to the identification of novel strategies to address conditions or disorders associated with muscle loss.

## 2. Skeletal Muscle 

**Figure 1:**
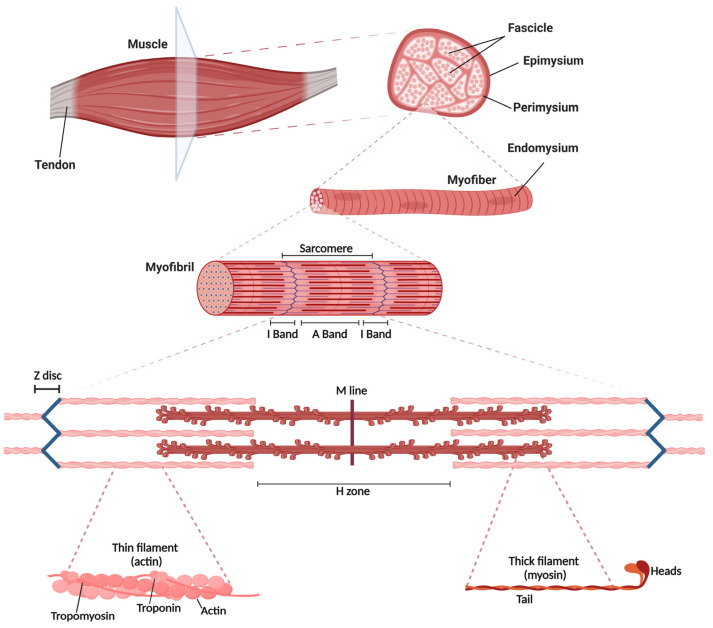
Structure of skeletal muscle, myofibers, and the sarcomere. The muscle is made up of several myofibrils packed into functional units surrounded by different layers of connective tissues (epimysium, perimysium and endomysium). One myofiber is composed of several sarcomeres, which is the main contractile unit mainly composed of protein filaments (myofilaments), namely myosin (thick filaments) and actin (thin filaments). The structure of the sarcomere presents dark and light bands, also visible with a light microscope. This is due to the alternated arrangement of the A bands (or anisotropic bands), the dark bands containing whole thick filaments, and the I bands (or isotropic bands), the light bands made up of only the thin filaments, located between two thick filaments. The Z disc across I bands demarcates the point of attachment between two adjacent actin filaments. The M line is considered the center of a sarcomere, whereas the H zone, which contains only myosin, is the area in between the M line and Z disc in resting conditions. Each myofibril is associated with troponin and tropomyosin, two regulatory proteins of muscle contraction.

Skeletal muscles are made up of myofibers, heterogeneous in type and biophysical characteristics, which are associated with each other in bundles surrounded by connective tissue anchored to bones through tendons (Figure 1). Myofibers are postmitotic multinucleated cells derived from the fusion of myoblasts during myogenesis. Each myofiber in humans has a size range of ~100 µm and is surrounded by a thin layer of connective tissue known as endomysium. The adjacent myofibers are therefore arranged in fascicles bounded by robust connective tissue, namely the perimysium. Each skeletal muscle is wrapped in an even stronger connective tissue membrane called the epimysium. The main role of these connective tissues is to preserve muscle and provide structural support during contraction. In addition, they connect muscle fibers with blood vessels and nerves. The plasma membrane of the myofiber, namely the sarcolemma, is in direct contact with the basal lamina, the material of the extracellular matrix. 

### 2.1. Skeletal Muscle Structure

Skeletal muscle has stripes that were first observed by the Dutch microscopist Antony van Leeuwenhoek (1632–1723) [3]. Each myofiber is made up of thousands of myofibrils composed of a series of sarcomeres, which represent the functional units responsible for muscle contraction. Each sarcomere is 2 μm in length and is mainly composed of myosin (thick filament) and actin (thin filament), which directly control muscle contraction. Muscle striations are the result of the regular arrangement of actin and myosin filaments. The sarcomere is composed of light I bands (isotropic bands) formed by thin filaments and darker A bands (anisotropic bands) formed by thick filaments located at the center of the sarcomere. The area of the A band in which the thick filaments are the only longitudinal elements under rest conditions is named the H zone. Each sarcomere is included between two consecutive dark lines called Z discs (or Z lines) to which the actin myofilaments are anchored. Myosin is the most abundant protein in muscle, roughly contributing for ~25% of the total amount of muscle proteins, and is the main driver of force generation. Each thick filament contains approximately 200 myosin molecules, each consisting of a rod-shaped region (coil-coil of two α-helices; Tail) with two heads, one of which is essential to bind adenosine triphosphate (ATP), and the other to bind one actin filament (Figure 1). 

Two large proteins, titin, and nebulin, ensure the correct alignment of the filaments. Titin spans from the Z disc to the center of the myosin filament, providing structural and elastic stabilization to the filaments. Furthermore, titin is responsible for the passive elasticity of the muscle after contraction [4,5,6]. Nebulin extends from the Z disc to near the tip of the thin filaments, thus regulating the length of the thin filament during sarcomere assembly. The interaction between actin and myosin is controlled by two regulatory proteins known as tropomyosin and troponin. Tropomyosin is a helical protein that is localized along the actin backbone and is fundamental to block the active site in actin and to inhibit the binding of actomyosin in the resting state. Troponin is a globular protein composed of three subunits (C, T, I) that direct its localization: troponin C has calcium (Ca^2+^) binding sites, troponin T binds to tropomyosin, and troponin I inhibits the binding of actin and myosin at rest. Tropomyosin and troponin inhibit muscle contraction when Ca^2+^ levels in the cytosol are low [7,8].

### 2.2. Skeletal Muscle Contraction

**Figure 2:**
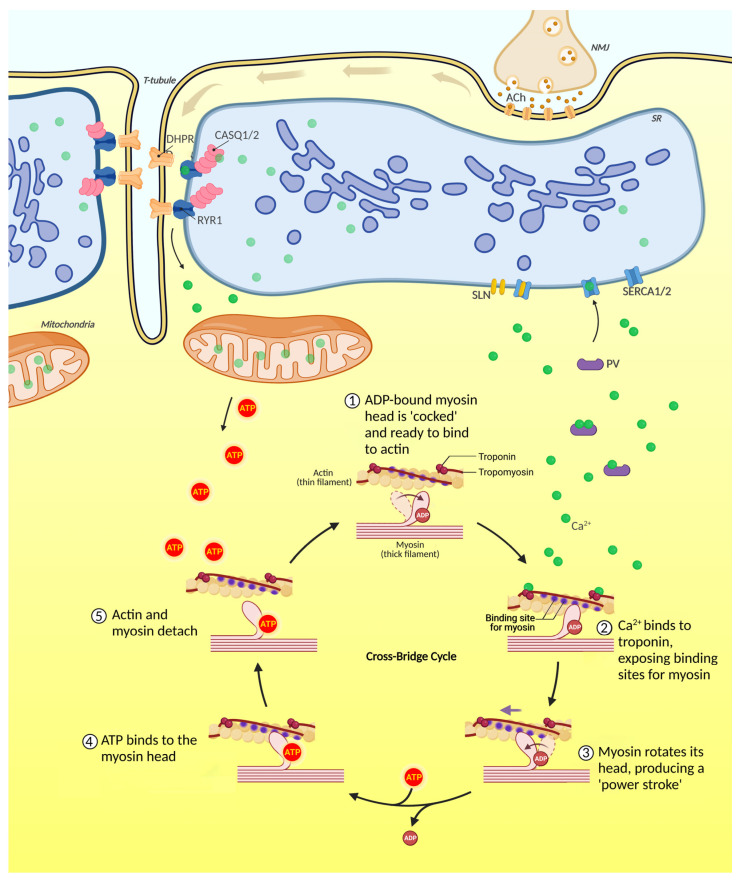
From neurotransmitter release to muscle contraction. The action potential running down to the motor axon invades the nerve terminal at the neuromuscular junction and triggers the fusion of the synaptic vesicles with the presynaptic membrane. Acetylcholine (ACh) is thus released and diffuses in the synaptic cleft to bind to the nicotinic ACh receptors expressed on the postsynaptic muscle fiber membrane. nAChRs are ionotropic ligand-gated Na^+^/K^+^ channels that cause a local depolarization of the muscle end-plate via inward flux of Na^+^. When the depolarization overcomes a critical threshold, voltage-gated Na^+^ channels open, thus triggering a post-synaptic action potential into the muscle fiber, which ultimately spreads out along the sarcolemma and invades the T-tubules (brown arrows). Here, an excitation-contraction molecular machinery transduces this electric signal into the cytosolic release of Ca^2+^ from the sarcoplasmic reticulum, leading to muscle fiber contraction.

An essential step to start muscle contraction is the release of Ca^2+^ by the sarcoplasmic reticulum (SR) in a process known as excitation-contraction coupling (ECC). This term was first adopted in 1952 by Alexander Sandow (1901–1978) to describe the rapid communication between electrical events arising in the plasma membrane and Ca^2+^ release, which ultimately lead to contraction [9]. The arrival of an action potential at the neuromuscular junction (NMJ), the specialized chemical synapse that the motor axon terminal forms with the myofiber triggers the release of acetylcholine, which diffuses along the sarcolemma and binds to its receptors (Figure 2).

When a critical amount of acetylcholine is released, the nicotinic acetylcholine receptors cause a local depolarization. Over a critical threshold, a post synaptic action potential propagates along the sarcolemma through the activation of voltage-gated Na^+^ channels. This process causes depolarization also at the “transverse tubules” (T-tubules), sarcolemma invaginations that insert between myofibrils. T-tubules are intimately associated with the SR in a region called terminal cisternae or junctional SR, and their membrane surface represents approximately 80% of the surface of the sarcolemma [10]. The high plasticity of T-tubules is fundamental for maintaining muscle stability during contraction and relaxation processes and facilitating repair after damage. A T-tubule with two terminal cisternae on both sides forms a particular structure known as the triad. In this region, depolarization activates the L-type Ca^2+^ channel Cav1.1 (dihydropyridine receptor, DHPR) in the sarcolemma. DHPR induces the opening of ryanodine receptors (RyR1) in the SR, resulting in a massive release of Ca^2+^ from the SR to the cytosol, thus triggering muscle contraction.

Ca^2+^ ions diffuse in the cytoplasm and bind to troponin C (Figure 2). Ca^2+^ binding to the troponin C subunit induces a rearrangement of the troponin complex with a consequent remodeling of tropomyosin, exposing active sites present in actin filaments. When the active sites are exposed, the myosin heads move toward the M-line, pulling actin along with it. Muscle contraction is an active and ATP-dependent process. The myosin heads in the active state are bound to adenosine triphosphate (ADP), which is released during contraction. ATP is required for cross-bridge detaching and is hydrolyzed when myosin returns to the initial position.

In the SR, Ca^2+^ is buffered by different Ca^2+^-binding proteins, namely calsequestrin 1 (CASQ1) and calsequestrin 2 (CASQ2). CASQ1 is expressed in both fast- and slow-twitch muscles, while CASQ2 (also known as cardiac CASQ) is present only in adult slow-twitch muscles. A molecule of CASQ1 can bind 80 Ca^2+^ ions, and the maximal Ca^2+^-binding capacity for CASQ2 is 60 ions [11,12]. The low level of free luminal Ca^2+^ concentration allows the transport of ions against a less steep gradient, causing a decrease in ATP hydrolysis and saving energy.

A key step to allow for another cycle of muscle contraction/relaxation is cytoplasmic Ca^2+^ clearance. Cytoplasmic Ca^2+^ is initially buffered by soluble cytoplasmic proteins, such as parvalbumin, and is finally removed from the cytosol by the Ca^2+^ ATPase of the SR (SERCA), mitochondria, and the Na^+^/Ca^2+^ exchanger, with fiber type-dependent kinetics. SERCA directs Ca^2+^ reuptake into the SR. Skeletal muscle expresses two isoforms of SERCA: SERCA1A is expressed mainly in fast glycolytic fibers, while SERCA2A is expressed primarily in slow-twitch and fast-oxidative fibers [13]. Furthermore, in recent decades, several proteins involved in the ECC process have been discovered, many of which have been linked to muscular disorders in humans [14]. 

### 2.3. Fiber Types and Metabolism

Since the first half of the nineteenth century, skeletal muscles have been classified based on their color as light or dark red, which was later found to be related to myoglobin levels, the speed of contraction, and the biochemical pathways they use to produce ATP. Using these criteria, skeletal muscle fibers can be divided into three main types: fast-twitch glycolytic fibers, slow-twitch oxidative fibers, and fast-twitch oxidative fibers. Fast-twitch glycolytic fibers have a lower mitochondrial content, rapid contraction kinetics, less dependence on oxidative phosphorylation (OXPHOS), and low fatigue resistance [15,16]. In the fast fibers, the hydrolysis of ATP occurs approximately twice as fast as in the slow fibers, which explains the faster kinetics of contraction. The fatigue is influenced by the amount of ATP. For these reasons, glycolytic fibers that produce ATP through anaerobic glycolysis fatigue more easily compared to oxidative fibers that rely on aerobic respiration. Slow-twitch oxidative fibers contain many mitochondria, show a strong dependence on OXPHOS, and can work for long periods without fatigue. Consequently, they play a key role in maintaining posture and producing small but frequent movements that do not require a huge amount of energy. Fast-twitch oxidative fibers present an intermediate phenotype; they contract relatively slowly and use aerobic respiration mainly to produce ATP. However, they can switch to glycolysis and fatigue more quickly than oxidative fibers [1]. Myosin ATPase hydrolyzes ATP to produce the cross-bridge that directly influences contraction speed. These fibers are used for frequent movements, among which walking, and require less energy than sprinting or jumping, but more energy than postural control.

Nowadays, we can identify four major fiber types in adult mammalian skeletal muscles based on the expression of different myosin heavy chain (MyHC) isoforms. Human limb muscles contain three isoforms of MyHC, namely—from the slower to the faster type—type I, type IIA and type IIX. A fiber can express a single myosin type or co-express different MyHC isoforms. Rodents express also another myosin isoform, the fastest, known as type IIB [1,17]. Muscle fibers could also be distinguished by mitochondrial content. In humans, for example, the mitochondrial volume is around 6% in type I fibers, 4.5% in type IIA fibers, and 2.3% in type IIX fibers. Differences in the number of mitochondria are an important factor because this modifies the thickness of the Z disc: sarcomeres rich in mitochondria, as those in slow soleus muscle fibers, have a thick Z disc, while sarcomeres with low mitochondrial content, as those in fast muscles, have a thin Z disc. Fatigue resistance is positively correlated with a large quantity of mitochondria and the thickness of the Z disc [18]. As mentioned above, muscle contraction is an ATP-dependent process. Although fast muscles require a rapid generation of ATP through glycolysis, slow muscles that normally do not require high levels of ATP leverage on mitochondrial oxidative phosphorylation. 

## 3. Contribution to the Special Issue

### 3.1. Section 1

The first section of this Special Issue is focused on pathological conditions that lead to muscle atrophy that may occur primarily in skeletal muscle or may be the consequence of diseases in other tissues and organs, including the central nervous system. The article entitled “*Master regulators of muscle atrophy: role of costamere components*”, by Luisa Gorza and colleagues [19], summarizes the variety of master regulators of muscle atrophy and how they are involved in different conditions leading to muscle atrophy. Although myofibril proteins and mitochondria have been extensively investigated in muscle atrophy development, the role of other macromolecular complexes, such as costameres, which connect the sarcolemma to the myofibrils and the extracellular matrix, is still under investigation. In this review, the authors focus on the early involvement of costamere component deregulation in the development of muscle atrophy.

Following the same theme, the review by Sandri et al. entitled “*Signaling in skeletal muscle atrophy: an organelle perspective*” addresses the role of organelles in muscle atrophy, ultimately summarizing the pathways involved, as well as the close relationship between organelle dysfunction and skeletal muscle atrophy. 

In the review entitled “*Metabolic pathways and ion channels involved in skeletal muscle atrophy: a starting point for potential therapeutic strategies*”, Ileana Canfora and colleagues have focused on metabolic pathways involved in skeletal muscle mass loss and atrophy, such as degradation, anabolic, and signaling pathways [20]. The authors also provide an overview of the potential drugs and natural compounds that counteract muscle mass loss and atrophy.

There is an increasing body of evidence that pathological processes occurring in skeletal muscle are important in diseases of the central nervous system, especially neurodegenerative diseases, including Alzheimer’s disease, Parkinson’s disease, polyglutamine diseases, and others. These diseases are characterized by progressive late-onset cognitive and motor symptoms associated with neuronal dysfunction and death, and protein misfolding and aggregation. The authors of this introductory paper present a review entitled “*Skeletal muscle pathogenesis in polyglutamine diseases*” [21]. An example of how peripheral tissues contribute to the progression and severity of symptoms in many neurodegenerative diseases is skeletal muscle, which is primarily or secondarily involved in the onset and progression of neurodegeneration. Here, the authors reviewed the role and contribution of skeletal muscle dysfunction to the pathogenesis of different polyglutamine diseases, discussing whether muscle could be a good therapeutic target to delay, if not stop, these harmful diseases.

### 3.2. Section 2

The second section of this Special Issue is focused on cancer cachexia, a syndrome that frequently develops in patients with cancer, congestive heart failure, chronic obstructive pulmonary disease, chronic kidney disease, and other conditions. It manifests itself as skeletal muscle atrophy, body weight loss, and progressive loss of lean body mass. There is currently no cure for cancer cachexia. In this issue, Giorgio Aquila and colleagues have nicely summarized the pathogenesis of cancer cachexia and the role of inflammation and oxidative stress. In their review entitled “*Nutraceuticals and Exercise against Muscle Wasting during Cancer Cachexia*” [22], the authors report recent evidence of a bimodal intervention, possibly more effective, involving both nutraceuticals and physical exercise at the same time to have a synergistic action in limiting cancer cachexia muscle wasting.

On the same line, Volker Adams and colleagues in their article entitled “*Small-Molecule Chemical Knockdown of MuRF1 in Melanoma Bearing Mice Attenuates Tumor Cachexia Associated Myopathy*” have used a mouse model of cancer cachexia to test two recently identified small molecules that inhibit MuRF1 activity, as well as MuRF1/MuRF2 expression [23]. Treatment improved muscle pathology and function and reduced body weight loss. The authors suggest that this strategy could decrease muscle atrophy and dysfunction in cancer cachexia. 

Several studies have reported that leucine supplementation can protect against hindlimb immobilization-driven atrophy. In this Special Issue, two research articles further support the beneficial effect of leucine supplementation on muscle atrophy. Laís Rosa Viana and colleagues contributed to the same theme with a paper entitled “*Leucine-Rich Diet Improved Muscle Function in Cachectic Walker 256 Tumour-Bearing Wistar Rats*” [24]. In this research article, the authors investigate the beneficial effects of a leucine-rich diet to prevent muscle atrophy in patients suffering from cachexia. Importantly, after functional analysis and evaluation of cachexia parameters, they found that although a leucine-rich diet alone could not completely reverse cachexia, it improved muscle strength and behavior performance in Walker 256 tumor-bearing rats.

Paula Alves and colleagues in the research article entitled “Leucine Supplementation Decreases HDAC4 Expression and Nuclear Localization in Skeletal Muscle Fiber of Rats Submitted to Hindlimb Immobilization” investigated what are the genes mediating this phenomenon and explored the histone deacetylase 4 (HDAC4)–leucine signaling axis [25]. They found that HDAC4 was highly expressed in the soleus muscle upon immobilization of the hindlimb and this effect was mitigated with leucine supplementation. Based on their findings, the authors suggest that the antiatrophic effects of leucine could be enforced by the inhibition of HDAC4. 

### 3.3. Section 3

The third section of this Special Issue is focused on treatments for skeletal muscle wasting and muscle atrophy after denervation and injury. NeuroHeal is a neuroprotective drug that was recently shown to enhance nerve regeneration and reduce muscle atrophy after nerve crush. In the research article entitled “*NeuroHeal reduces muscle atrophy and modulates associated autophagy*”, Marmolejo-Martínez-Artesero and colleagues uncover the direct role of this therapeutic agent in muscle biology [26]. They found that NeuroHeal can reduce muscle atrophy in different models, both in vivo and in vitro, by promoting a reduction in proteasomal activity and restoring autophagy flux, with a mechanism likely to involve SIRT1 activation. Following these findings, the same group headed by Caty Casas has further investigated the potential of NeuroHeal as a treatment for injured muscles and has demonstrated that it improves muscle fiber recovery while significantly reducing scar tissue formation, as reported in the research article entitled “*NeuroHeal improves muscle regeneration after injury*” [27]. Taken together, the authors suggest that NeuroHeal could be a valuable novel treatment for different types of disorders. 

On the same line with regards to novel potential therapeutic targets, Jinwoo Lee and colleagues in the paper “*Protein arginine methyltransferases in neuromuscular function and diseases*” focused on the involvement of protein arginine methyltransferases (PRMTs) in the pathophysiology of neuromuscular diseases [28]. There is a critical need for new treatments to stop these disorder, for which a cure is currently lacking. This is largely because the cause of many neuromuscular diseases is still unclear. Therefore, many efforts have been focused on the investigation of putative players that could exert important roles in the maintenance of muscle health. Post-translational modifications are crucial for the achievement of many fundamental biological processes, spanning from gene transcription to signal transduction. Here, the authors focus on PRMTs, detailing their role in muscle homeostasis and remodeling, neuromuscular function, and neuromuscular diseases, ultimately providing the rationale for considering PRMTs as a valuable target for treatment. 

Laura Cussonneau and colleagues focused the paper entitled “Concurrent BMP signaling maintenance and TGF-β signaling inhibition is a hallmark of natural resistance to muscle atrophy in the hibernating bear” on the identification of novel drivers for muscle atrophy resistance, paving the way to the identification of new potential candidates for treatment [29]. To this aim, the authors took advantage of a model that is naturally resistant to muscle atrophy upon fasting and disuse, the hibernating brown bear. They focused on TGF-β and BMP intracellular pathways, which are involved in muscle mass loss and maintenance, respectively, and found that hibernating brown bears hold a balance between these two pathways to prevent atrophy. In particular, atrophy-resistant muscles showed a down-regulation of the TGF-β pathway, with a concomitant up-regulation of the BMP pathway. These findings provide the basis for a novel therapeutic strategy that aims to adjust the BMP pathway in TGF-β inhibiting therapies to maintain balance, ultimately preventing disuse-induced muscle atrophy.

Skeletal muscle wasting is also a detrimental feature in diseases in which muscles are not a primary target tissue, such as type 1 diabetes. In the paper entitled “*Schisandrae chinensis fructus extract ameliorates muscle atrophy in streptozotocin-Induced diabetic mice by downregulation of the CREB-KLF15 and autophagy–lysosomal Pathways*” Ho-Jung Choi and colleagues have investigated the beneficial effects of Schisandrae chinensis fructus (SFe) extract on muscle wasting [30]. They found that although SFe did not alter the blood glucose levels in streptozotocin (STZ)-induced diabetic mice, it increased muscle weight, cross-sectional area, and muscle strength. The authors investigated several pathways involved in atrophy and autophagy, and ultimately found that SFe decreases protein degradation by down-regulating the CREB-KLF15-mediated UPS system and the p62/SQSTM1-mediated autophagy–lysosomal pathway. Choi and colleagues ultimately suggest SFe as a treatment for muscle wasting in type 1 diabetes. 

Yufang Liu and colleagues in the paper entitled “VMP1 Regulated by chi-miR-124a Effects Goat Myoblast Proliferation, Autophagy, and Apoptosis through the PI3K/ULK1/mTOR Signaling Pathway” advanced our knowledge of the mechanism involved in myoblast proliferation, autophagy, and apoptosis [31]. A complete understanding of the regulatory mechanisms that underlie muscle development is essential in the development of novel therapeutic interventions. They have identified VMP1 as the promoter of myoblast proliferation and autophagy, while inhibiting apoptosis. The authors have also found that VMP1 expression in myoblasts is regulated by miR-124a, and it affects autophagy by targeting the ULK1/mTOR pathway.
